# CT perfusion in peripheral arterial disease—hemodynamic differences before and after revascularisation

**DOI:** 10.1007/s00330-021-07692-5

**Published:** 2021-02-06

**Authors:** Patrick Veit-Haibach, Martin W. Huellner, Martin Banyai, Sebastian Mafeld, Johannes Heverhagen, Klaus Strobel, Bert-Ram Sah

**Affiliations:** 1 Department of Nuclear Medicine, University Hospital Zurich, Zurich, Switzerland; 2 Department of Radiology, University Hospital Zurich, Zurich, Switzerland; 3 University of Zurich, Zurich, Switzerland; 4 Joint Department of Medical Imaging, University of Toronto, Toronto, Canada; 5 Department of Radiology and Nuclear Medicine, Lucerne Cantonal Hospital, Lucerne, Switzerland; 6 Department of Internal Medicine, Subdivision of Angiology, Lucerne Cantonal Hospital, Lucerne, Switzerland; 7 Clinic for Angiology, University Hospital Zurich, Zurich, Switzerland; 8 Department of Diagnostic, Interventional, and Pediatric Radiology, Inselspital, University of Bern, Bern, Switzerland; 9 Department of Cancer Imaging, King’s College London, London, UK

**Keywords:** Peripheral arterial disease, Perfusion imaging, Volumetric CT, Contrast media

## Abstract

**Objectives:**

The purpose of this study was the assessment of volumetric CT perfusion (CTP) of the lower leg musculature in patients with symptomatic peripheral arterial disease (PAD) before and after interventional revascularisation.

**Methods:**

Twenty-nine consecutive patients with symptomatic PAD of the lower extremities requiring interventional revascularisation were assessed prospectively. All patients underwent a CTP scan of the lower leg, and hemodynamic and angiographic assessment, before and after intervention. Ankle-brachial pressure index (ABI) was determined. CTP parameters were calculated with a perfusion software, acting on a no outflow assumption. A sequential two-compartment model was used. Differences in CTP parameters were assessed with non-parametric tests.

**Results:**

The cohort consisted of 24 subjects with an occlusion, and five with a high-grade stenosis. The mean blood flow before/after (BFpre and BFpost, respectively) was 7.42 ± 2.66 and 10.95 ± 6.64 ml/100 ml*min^−1^. The mean blood volume before/after (BVpre and BVpost, respectively) was 0.71 ± 0.35 and 1.25 ± 1.07 ml/100 ml. BFpost and BVpost were significantly higher than BFpre and BVpre in the treated limb (*p* = 0.003 and 0.02, respectively), but not in the untreated limb (*p* = 0.641 and 0.719, respectively).

**Conclusions:**

CTP seems feasible for assessing hemodynamic differences in calf muscles before and after revascularisation in patients with symptomatic PAD. We could show that CTP parameters BF and BV are significantly increased after revascularisation of the symptomatic limb. In the future, this quantitative method might serve as a non-invasive method for surveillance and therapy control of patients with peripheral arterial disease.

**Key Points:**

*• CTP imaging of the lower limb in patients with symptomatic PAD seems feasible for assessing hemodynamic differences before and after revascularisation in PAD patients.*

*• This quantitative method might serve as a non-invasive method, for surveillance and therapy control of patients with PAD.*

**Supplementary Information:**

The online version contains supplementary material available at 10.1007/s00330-021-07692-5.

## Introduction

Peripheral arterial disease (PAD) is a common vascular disease, which affects approximately 237 million people worldwide, with a rising global prevalence [1, 2]. Intermittent claudication and ulcers are frequent symptoms [3, 4]. The ankle-brachial index (ABI) is a well-established indicator for PAD, and commonly used for clinical evaluation of symptomatic patients. To date, invasive subtraction angiography is considered the standard of reference in vascular imaging [5]. It is used to detect the site of vascular stenosis or occlusion and allows for therapeutic revascularisation in the same procedure. Computed tomography angiography and magnetic resonance angiography recently saw huge technical improvement and were introduced into current guidelines [5–8].

However, the causative lesion for acute symptoms does neither reflect the entire disease burden nor the degree of functional collateralisation. Furthermore, since PAD is a generalised disease, alterations of the microvascular density might change the vascular supply of tissue significantly. On the one hand, it was shown that in limbs with a severe stenosis (and subsequent hypoxia), ischemia induces neo-angiogenesis [9]. On the other hand, microvascular occlusion might decrease the perfusion of musculature. Hence, there is an elementary need for a technique that provides observer-independent, reproducible, and reliable quantitative assessment of blood perfusion in extremities. Such a method should ideally not only display the morphological surrogate that is partly the reason the symptoms of the patients but reflect the whole extent of disease. Such a method cannot be exclusively based upon the blood stream in the largest vessels of the extremities, which are the basic underlying physiologic parameters of the ABI [5, 10].

Recently, efforts were made to investigate PAD using volumetric computed tomography perfusion (CTP) [10–12]. CTP parameters were shown to reflect microvessel density in tumours and other different human tissues [13, 14]. They might serve as a more holistic assessment tool of vascular disease, complementing other modalities. Therefore, the purpose of our study was the assessment of volumetric CTP of the lower leg musculature in patients with symptomatic PAD before and after interventional revascularisation, in order to further characterise the potential role of this non-invasive method for the quantitative in vivo assessment of limb ischemia.

## Materials and methods

This prospective study was approved by the institutional review board and by the cantonal ethics committee (Kantonale Ethikkommission Luzern, No. 1014). All patients provided informed signed consent prior to the examinations. Twenty-nine consecutive patients (median age 72 years, range 48 to 87 years, 13 females, 22 males) with symptomatic PAD of the legs were evaluated. All patients were referred for angioplasty, in particular for unilateral interventional revascularisation of arteries that provide blood supply to the lower leg. Symptomatic PAD was defined as prevalence of intermittent claudication, ischemic rest pain and ischemic tissue loss, such as gangrene or non-healing ischemic ulcers, and ultrasonographic evidence of a hemodynamically significant obstruction of the common iliac, external iliac, or superficial femoral artery. Only patients with unilateral evidence of hemodynamically significant obstruction were included in this study. Medical history was recorded. Further details are given in S1 File.

All subjects underwent clinical assessment of hemodynamic parameters within 24 h prior to revascularisation, and again immediately after the intervention (see below). Angiographic parameters were assessed at the beginning of the interventional revascularisation. Immediately before and after the revascularisation procedure, all patients underwent a CTP scan of the lower leg.

### Hemodynamic assessment

Hemodynamic assessment was performed as previously described in all patients prior to and after revascularisation [10]. Evidence of hemodynamically significant obstruction was established by non-invasive vascular testing, pulse volume recordings, and duplex ultrasound imaging studies. Maximum ankle-brachial pressure index (ABI) was recorded in a standardised way as reported previously [10]. Further details are given in S1 File.

### CTP assessment

All CTP examinations were performed as previously described [10]: A 128-slice CT scanner (Somatom Definition Flash, Siemens Healthineers) was used in shuttle mode, allowing for 28.2 cm of axial coverage and 2-s temporal resolution, which included almost all muscles of both lower legs in every subject. After placement on the gantry table, patients’ feet were fixated using tape to prevent spontaneous movement. The tube current was set to 100 mAs, the tube voltage to 100 kV(p). The duration of the CT perfusion scan was 60 s, with a rotation time of 2 s. CTP was started 15 s after injection of 60 ml of contrast medium (CM) (Ultravist 370, Bayer AG) at 5 ml/s. CM was injected into an antecubital vein by a dual-head pump injection device (Stellant D, Medrad), followed by a flush of 50 ml of NaCl at 5 ml/s. The collimation was 64 × 0.6 mm. The reconstruction increment was 10 mm at 10 mm slice width. Image reconstruction was performed with a 512 × 512 pixel matrix and medium smooth B30f kernel. For image post-processing and analysis, the reconstructed images of both legs were transferred to a commercially available computer workstation (Syngo Multimodality Workplace, Siemens Healthineers).

CTP parameters blood flow (BF; ml/100 ml*min^−1^), blood volume (BV; ml/100 ml), and mean transit time (MTT; s) were calculated with a perfusion software (Syngo Volume Perfusion CT Body, Siemens Healthineers), based on the Patlak analysis. Since veins did not show sufficient CM enhancement in the late phase of the dynamic series and since venous enhancement was furthermore often degraded by pathologic flow within varicose veins in our patient cohort, no outflow curve was obtained. Thus, as previously described in the literature, we acted on a no outflow assumption [11, 15–17]. Dataset motion correction and a noise reduction algorithm were applied automatically. Processing thresholds or segmentation tissue limits were − 50 HU and + 150 HU to exclude bone, vessel wall calcification, and other hyperdense material. Window width and centre for reference vessel input was + 300 HU and + 150 HU, respectively. The relative threshold for inside and outside was 50%, an adaptive smoothing filter was used. The vendor’s standard algorithmic parameters were applied. 3D colour-coded maps for BF, BV, and MTT were generated with a sequential two-compartment model. BF is defined as the amount of blood flowing through 100 ml of muscle tissue within 1 min. MTT is defined as the average time of contrast agent residence within the muscle tissue. BV is defined as the amount of blood within 100 ml of muscle tissue. BV can be expressed as proportion of the total volume of a dedicated voxel. For every patient, an individual arterial input function was determined by placing a region of interest (ROI) into the popliteal artery. A dedicated free-hand user-defined ROI was drawn around the lower leg muscles on every slice and adapted to their confines. Adjacent bones, vessels, and other soft tissue structures were excluded. All image evaluations were performed by two experienced radiologists in consensus with 8 and 12 years of experience.

### Angiographic assessment

Before the start of revascularisation, the length of the occlusion or stenosis was measured and classified on subtracted angiographic images (Allura Xper FD, Philips), as reported previously [10]. The degree of collateralisation was assessed by visual estimation on angiographic images. It was rated as good (1) if the estimated cross-sectional area of collaterals added up to > 50% of the pre-lesion diameter of the collateralized vessel, medium (2) if < 50%, and poor (3) if only faint or no collaterals were visible. The performing interventional radiologist had 20 years of experience in the field of revascularisation procedures.

### Statistical analysis

Demographic, hemodynamic, angiographic, and CTP parameters in patients with stenosis and occlusion were compared by Mann-Whitney *U* test. Differences of CTP parameters between side of hemodynamically significant obstruction (treated limb), and the other side (untreated limb), as well between pre- and post-revascularisation were analysed by Wilcoxon signed-rank test. The correlation between CTP, hemodynamic, and angiographic parameters was analysed using Spearman’s correlation coefficient. Results were interpreted as almost perfect correlation between ± 0.81 and ± 1.00, substantial between ± 0.61 and ± 0.80, moderate between ± 0.41 and ± 0.60, fair between ± 0.21 and ± 0.40, thereunder no correlation [18]. A *p* value of < 0.05 was considered statistically significant. All analyses were performed using IBM SPSS Statistics™ 25.0.0 (IBM).

## Results

### Patients and clinical parameters

Twenty-four (83%) of our 29 patients (71 ± 11.37 years old, range 48–87 years) were referred for interventional revascularisation of an occlusion, five patients (17%) for a high-grade stenosis. The mean length of the lesion was 8.31 ± 7.62 cm (range 1–25). All lesions were located unilaterally in the common iliac, external iliac, or superficial femoral artery. ABP values indicative of media sclerosis were found in 6 subjects (5 occlusions, 1 stenosis). ABP and ABI values of these subjects were hence excluded from further analysis. Five patients had poor, 14 medium, and 10 patients a good degree of collateralisation.

### Comparison of CTP parameters before and after revascularisation

**Fig. 1:**
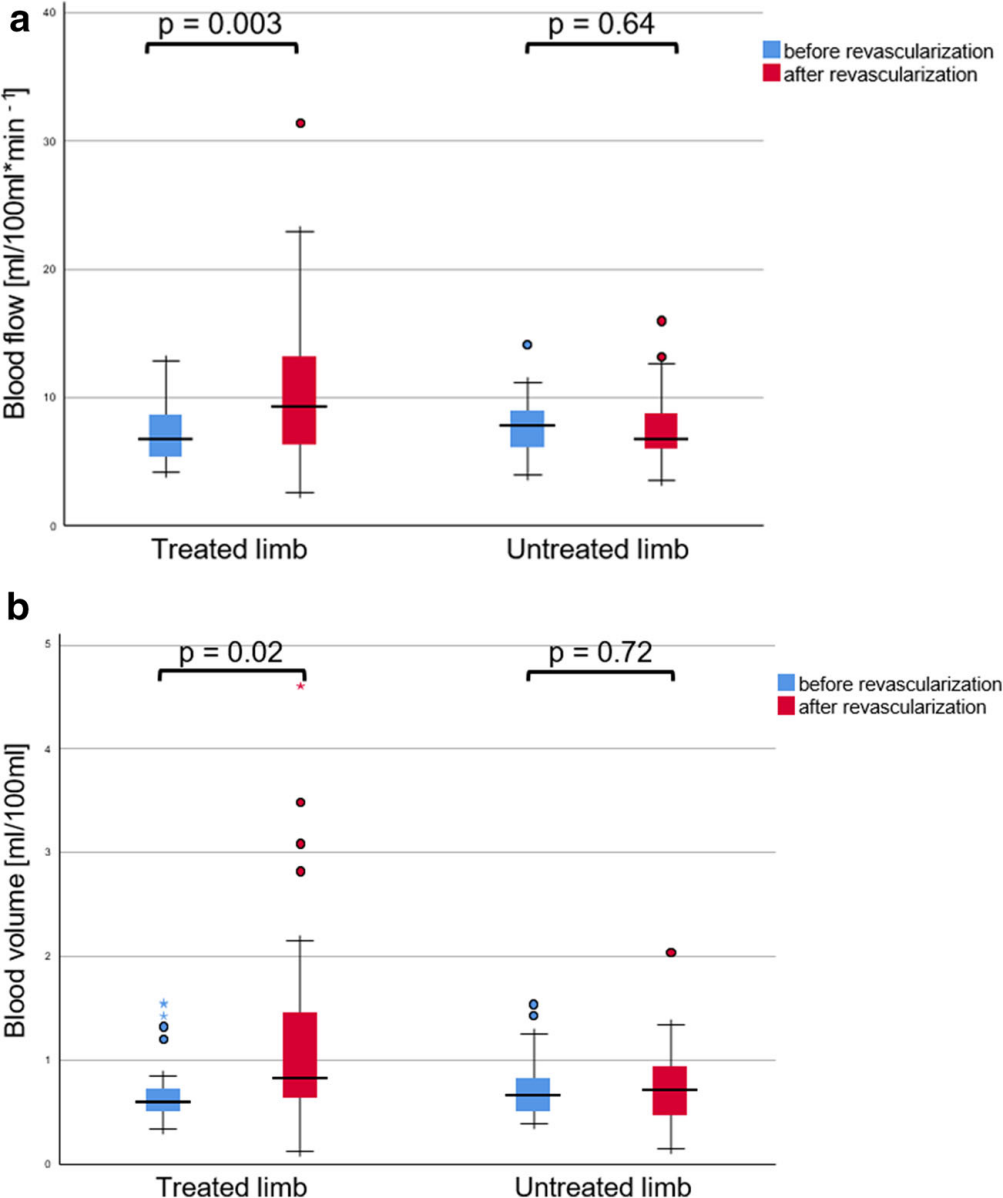
**a** Blood flow before and after revascularisation. **b** Blood volume before and after revascularisation

The mean blood flow before and after revascularisation (BFpre and BFpost, respectively) was 7.42 ± 2.66 and 10.95 ± 6.64 ml/100 ml*min^−1^, respectively (Fig. 1a). The mean blood volume before and after revascularisation (BVpre and BVpost, respectively) was 0.71 ± 0.35 and 1.25 ± 1.07 ml/100 ml, respectively (Fig. 1b). Post-intervention values of blood flow, blood volume, ABI, and ABP were significantly higher than respective pre-intervention values in the treated limb (*p* = 0.003, 0.02, < 0.001, and < 0.001, respectively), but not in the untreated limb (*p* = 0.64, 0.72, 0.36, and 0.57, respectively) (Table 1). ABI, ABP, and the degree of collateralisation were not correlating to any blood flow or blood volume parameter (*p* > 0.15 for all correlations).

**Table 1:** mean ± SD values

*n*	CT perfusion and clinical parameter	Treated limb	Untreated limb	*p* value
29	BF pre [ml/100 ml*min^−1^]	7.42 ± 2.66	7.70 ± 2.45	0.76
29	BF post [ml/100 ml*min^−1^]	10.95 ± 6.64	7.52 ± 2.98	< 0.001
29	BV pre [ml/100 ml]	0.71 ± 0.35	0.73 ± 0.30	0.97
29	BV post [ml/100 ml]	1.25 ± 1.07	0.75 ± 0.39	< 0.001
23	ABI pre	0.60 ± 0.18	0.87 ± 0.19	< 0.001
23	ABI post	0.95 ± 0.25	0.94 ± 0.28	0.89
23	ABP pre [mmHg]	78.26 ± 29.53	126.22 ± 46.47	< 0.001
23	ABP post [mmHg]	132.88 ± 44.46	134.42 ± 48.48	0.60

*ABI*, ankle-brachial pressure index; *ABP*, ankle blood pressure; *pre*, before revascularisation; *post*, after revascularisation

## Discussion

Our study shows post-interventional quantitative CTP imaging to be feasible for assessing blood perfusion of the lower limb in patients with PAD. To our knowledge, this is one of the earliest reports evaluating pre- and post-interventional CTP parameters of the lower leg in clinical situations requiring interventional treatment. Furthermore, our study analyses the correlation of CTP parameters with established clinical parameters.

We could show that BF and BV were significantly increased after revascularisation of the treated limb. In the untreated limb without acute symptoms, no significant difference was found after revascularisation of the contralateral side. These findings are assumed to be driven by the higher overall cross-sectional vascular diameter on the side of the acute symptomatic vessel stenosis or occlusion, resulting from the reopened vessel in addition to the pre-existing collaterals [10]. Furthermore, our study shows no significant differences of pre-interventional CTP parameters between the treated and untreated side. This finding is likely explained by the acquisition of scans during a rest situation. However, in more severe disease (i.e. with lower average ABI), one would expect to see a difference. Additionally, it is well-known from clinical symptoms and from cardiac perfusion scans, ischemic muscle appears normally at stress. However, after intervention, significantly higher perfusion values were found in the treated limb in comparison to the untreated limb without acute symptoms. This measured increase in perfusion is likely explained by additional blood flow and volume admitted by the reopened major artery in conjunction with inflow through pre-existing collaterals. These collaterals were formed previously due to significant ischemia, and maintained blood distribution to the dependent musculature unto a fragile equilibrium, which manifested clinically as claudication or even critical limb ischemia. At this point, collaterals were presumably dilated to a maximum, and responsiveness to any intrinsic vasoconstrictor stimuli was minimised also at rest.

Interestingly, the well-established clinical parameters ABI and ABP showed partly contrary behaviour, with significantly lower values in the treated and symptomatic limb than in the untreated limb before intervention, and no difference between both limbs after intervention. Both parameters reflect systolic blood pressure in the main arteries. They do neither account for regional blood supply nor for microvascular disease, and probably also not for smaller collateral vessels. Therefore, not surprisingly, CTP parameters were not correlated to these clinical parameters in our study. BF and BV on the other hand are complex parameters, which seem to target different hemodynamic factors. They take into account other important aspects and may contribute to the understanding of the complexity of PAD as a generalised disease: the blood supply via collaterals, as well as dilated microvessels, neo-angiogenesis, and micro-occlusion within the muscular compartment. Their value was proven on a histopathologic basis [13, 14]. Directly after intervention, CTP parameters might be slightly overestimated because of post-ischemic vasodilatation and permeability.

CTP parameters are presumably directly linked to the supply of tissue with immune cells and nutrients, as well as with oxygen and the washout of metabolic products, all of which need a functioning microcirculation [19]. These imaging parameters might be better correlating to classical symptoms such as pain, which represents the simplest clinical indicator of tissue ischemia. Also, pain evaluation, even when measured with scaling questionnaires, is somewhat subjective and variable. Decreasing blood perfusion values could help indicate the adequate time for re-intervention before another acute event occurs.

A group around Gao et al found in their CTP study overall promising results [12]. They evaluated the change of blood supply of the foot with CTP before and after endovascular treatment in 19 patients with PAD. On the treated side, blood flow increased significantly after intervention. In contrast to our study, they did not observe a significant increase in blood volume. Possible reasons for this difference might be the lower number of patients in their study, and the much smaller volume of tissue investigated in the foot, in contrast to the larger ROI in lower leg muscles in our study. We expect perfusion values obtained in lower leg muscles to be more robust, since they are more independent of ambient temperature and partly more resistant to movement artifacts, if patients are positioned properly [20]. In contrast to the abovementioned study, we did not have to exclude patients because of movement during the scan. Also, ischemic pain, which represents an intermediate finding during the course of disease, is typically elicited in the lower leg, not necessarily in the foot, despite the fact that the foot represents a more distal point of vascular supply, and hence is also more prone to ulcers, which is a late finding. Further studies comparing perfusion measurements at different levels of the leg intra-individually might be useful to gain more information about a possible lower leg hypoxia/blood supply gradient. Such studies will be able to account for regional differences in perfusion, and might help stratify tissue at risk for ulcers. Besides proximal vessel pathology, other factors such as chronic downstream microvascular occlusion are limiting the lifetime of the dependent tissue, and this complex situation is not represented by simple, large vessel-based parameters [10].

Recapitulating our results, we have shown that CTP values obtained in calf musculature, which represents the anatomic substrate for the clinical hallmark of critical limb ischemia, i.e. claudication, might represent an appropriate overview of local PAD [10]. CTP accounts for physiological compensation mechanisms of the body and functional collateralisation. It might therefore be a suitable tool for PAD patients, completing the diagnostic workup, and furthermore, it might play a role in monitoring patients under treatment. ABI and ABP on the other hand focus on blood flow in major arteries. Whereas both, CTP and clinical parameters as ABI and ABP, revealed significant improvement of perfusion before and after revascularisation of the treated limb, clinical parameters proved its value in depicting the side with acute symptoms before intervention better than CTP parameters. CTP on the other hand revealed significant differences between both sides after revascularisation.

Some limitations of our study deserve to be mentioned. We did perform consensus reading of results [21]. Patients with stenosis and occlusions were not evaluated separately due to the low number of individuals in the stenosis subgroup. Patients were scanned at rest, and not at stress. The latter situation might provide additional insights into vasculature function, but can hardly be achieved in a standardised way with patients positioned on the gantry table of a CT scanner. In addition, we did not obtain perfusion data in healthy subjects for comparison. This data would however be required to be age-matched (and any elderly subject without symptoms cannot simply be considered as “vascular healthy”). Therefore, it is ethically not justifiable to acquire this data in volunteers within the scope of this early clinical research.

We did not evaluate catheter-derived pressure gradient measurements across the treated vascular lesion. Mean arterial pressure gradients would be interesting to compare, however possibly not correlating, because they do not take into account other factors as microvascular obstruction or collateralisation. Furthermore, for considering of different grades and sites of possible PAD stenosis and occlusions, a larger patient cohort would be necessary for this comparison. Historically, pressure gradients have been a useful tool in iliac angiography to determine lesion significance but their role is less well understood for the peripheral vasculature [22]. From a theoretical standpoint, it would have been interesting to know the trans-lesional pressure gradients in this study; however, given the variations in reported techniques in the literature, this data may confound interpretation. For example, Garcia et al demonstrated that catheter-based pressure gradients may overestimate the true gradient compared with a pressure wire [23]. Furthermore, Banerjee et al have suggested that vasodilation therapy is a useful adjunct to understand the hemodynamic significance of trans-lesional pressure gradients, but standardised approaches to integration for peripheral vascular intervention are less established compared with endovascular coronary intervention [24, 25].

## Conclusions

CTP is feasible for assessing hemodynamic differences in calf muscles before and after revascularisation in patients with symptomatic PAD. We could show that CTP parameters BF and BV are significantly increased after revascularisation of the symptomatic limb. In the future, this quantitative method might serve as a non-invasive method for surveillance and therapy control of patients with peripheral arterial disease.

## Supplementary Information

ESM 1 **S1 File**: Further details of recorded data, inclusion criteria and hemodynamic assessment are given in the supporting information file. (DOCX 18 kb)

## Abbreviations

3D: Three-dimensional
4D: Four-dimensional
ABI: Ankle-brachial pressure index
ABP: Ankle blood pressure
BF: Blood flow
BV: Blood volume
CM: Contrast medium
CT: Computed tomography
CTP: CT perfusion
CW: Continuous wave
HU: Hounsfield unit
MIP: Maximum intensity projection
MRI: Magnetic resonance imaging
MTT: Mean transit time
PACS: Picture archiving and communication system
PAD: Peripheral arterial disease
PSV: Peak systolic velocity
*r*: Correlation coefficient
*r*^2^: Correlation coefficient of determination
ROI: Region of interest
SD: Standard deviation
SPO: Segmental pulse oscillography
US: Ultrasound
VOI: Volume of interest
